# Zinc and Iron Nutrition Status in the Philippines Population and Local Soils

**DOI:** 10.3389/fnut.2019.00081

**Published:** 2019-06-07

**Authors:** Alvin D. Palanog, Mark Ian C. Calayugan, Gwen Iris Descalsota-Empleo, Amery Amparado, Mary Ann Inabangan-Asilo, Emily C. Arocena, Pompe C. Sta. Cruz, Teresita H. Borromeo, Antonio Lalusin, Jose E. Hernandez, Cecilia Acuin, Russell Reinke, B. P. Mallikarjuna Swamy

**Affiliations:** ^1^Strategic Innovation Platform, International Rice Research Institute, Los Baños, Philippines; ^2^College of Agriculture and Food Science, University of the Philippines, Los Baños, Philippines; ^3^PhilRice Negros, Philippine Rice Research Institute, Science City of Muñoz, Philippines; ^4^College of Agriculture, University of Southern Mindanao, Kabacan, Philippines

**Keywords:** biofortification, iron deficiency, micronutrients, rice, zinc deficiency

## Abstract

The Philippines is one of the major rice-producing and rice-consuming countries of Asia. A large portion of its population depends on rice for their daily caloric intake and nutritional needs. The lack of dietary diversity among poor communities has led to nutritional consequences, particularly micronutrient deficiencies. Iron-deficiency anemia (IDA) and zinc deficiency (ZnD) are two serious nutritional problems that affect the health and economic sector of the country. Since rice dominates the Filipino diet by default, biofortification of rice will help improve the micronutrient status. The Philippine government has proactively initiated various programs and policies to address micronutrient deficiencies, particularly through fortification of basic food commodities. Biofortification, the fortification of rice with micronutrients through breeding, is considered the most sustainable and cost-effective strategy that can benefit large vulnerable populations. However, developing promising genotypes with micronutrient-enriched grains should be coupled with improving micronutrient bioavailability in the soil in order to optimize biofortification. This review documents the prevailing soil Zn-deficiency problems in the major rice production areas in the Philippines that may influence the Zn nutritional status of the population. The article also reports on the biofortification efforts that have resulted in the development of two biofortified varieties approved for commercial release in the Philippines. As nutritional security is increasingly recognized as a priority area, greater efforts are required to develop biofortified rice varieties that suit both farmers' and consumers' preferences, and that can address these critical needs for human health in a sustainable and cost-effective manner.

## Introduction

Rice is the most important staple food consumed by more than half of the world's population. Unfortified modern rice varieties with small amounts of micronutrients in the grains supply only a fraction of the daily individual requirements. Dependence on rice as the major dietary source of micronutrients contributes to micronutrient deficiency.

The Philippines accounts for 2.8% of global rice production and is the eighth largest rice producer in the world (1). The annual per capita consumption of rice in the country is estimated at 109.5 kg, which provides 56% of the calories, 37% of the proteins, and 3.8% of the fats required per person per day (2). However, most high-yielding and popular varieties currently grown are poor sources of micronutrients such as iron (Fe) and zinc (Zn), especially in their polished form (3, 4), and contain an average of 2 ppm of Fe and 12 ppm of Zn (5). Meanwhile, brown rice contains 6.3–24.4 ppm of Fe and 13.5–28.4 ppm of Zn. The endosperm, bran, and hulls of rice grain contain about 34.6, 27.0, and 38.4% of Fe and 56.6, 23.8, and 19.6% of Zn, respectively (6). The amounts of these micronutrients decrease after polishing (6).

The daily Zn requirement of an adult individual ranges from 8 to 11 mg per day while pregnant and lactating women require the highest intakes at 11–13 mg per day (7). The daily Fe requirement in adults varies from 12 to 28 mg per day while pregnant and lactating women require 30–38 mg per day (8). Current food intakes are unable to meet these requirements. Iron supplements are therefore provided to pregnant and lactating women, and zinc supplements are part of child diarrhea management. Iron fortification has been mandated by law for rice and flour (more on this below). But these efforts are insufficient as at present micronutrient malnutrition affects a large portion of the Philippine population (9) and is an important public health concern domestically.

Iron-deficiency anemia (IDA) is the most prevalent micronutrient deficiency affecting a large proportion of infants (40.5%), pregnant women (26.4%), lactating women (16.7%), and elderly males (23.0%) in the country. Anemia can cause profound impact on human health and productivity and Fe deficiency has pronounced effects on the first 1,000 days of life (10).

Zinc deficiency (ZnD) is also prevalent in 25.6% of the population based on serum Zn level tests. The elderly >60 years old (36.3%), adults 20–59 years old (28.1%), and lactating women (25.2%), followed by adolescents (23.6%), and school children (21.6%), are at high risk for Zn deficiency among the different populations and physiological groups in the country (2). ZnD contributes to impaired immune systems and slow child growth.

In the Philippines, rice is seen as an effective delivery system for food fortification because of its high per capita consumption (109 kg/person/day). An increment in micronutrient content in rice would positively impact human nutrition and health (11). Food fortification is considered a cost-effective approach in increasing the micronutrient content of rice although it poses several disadvantages: fortified foods should be consumed in an adequate amount, they should not affect the sensory properties of foods, and they need a safe delivery system and continued investments (12, 13). A more cost-effective and sustainable approach for rice fortification is biofortification. It is a process of increasing the concentrations of bioavailable micronutrients in the edible parts of the plant by traditional breeding, genetic engineering, or agronomic approaches (14).

Conventional plant breeding and genetic engineering are means of improving the genetic traits of crops to enhance the accumulation of bioavailable micronutrients (15). Both approaches have proven effective in increasing Zn content while genetic engineering is more effective in improving Fe content. Conventional plant breeding is a time-consuming process and its effectiveness is influenced by the availability of micronutrients in the soil (16). Thus, it is important to consider the various soil or edaphic factors and management options that improve Fe and Zn soil availability, which influences the accumulation of micronutrients in biofortified rice.

In this review, we briefly discuss rice production and dietary consumption in the Philippines and the importance of Fe and Zn in the health of Filipinos. This article highlights the micronutrient deficiency status of the Philippines soil and Filipino population and their possible association. It also gives an overview of the progress in rice biofortification efforts in the country and the programs and interventions that are implemented by the government to address micronutrient deficiency problems.

## Rice is an Important Component of the Filipino Diet and an Efficient Vehicle for Fortification

The Philippines is a rice-producing and -consuming country. The area allotted for rice production in the country was about 4.81 million hectares in 2017 (17), the majority of which was irrigated (69.7%), followed by rainfed (30.0%), and the rest was upland and saline-prone areas (18). Majority of the rice farmers use high quality inbred rice seeds (45%), farmers good seeds (FGS) {43%}, hybrid seeds (9%) and lastly, traditional varieties (3%). Highest volume produced was from inbred rice with 8.3 million metric tons (mmt) followed by hybrid (2.08 mmt), FGS (0.16 mmt), and traditional varieties (0.06 mmt). Largest area was planted with FGS (2.5 million hectares) followed by inbred seeds (~2.0 million hectares) and both hybrid and traditional varieties were planted to < 1 million hectares (17). Rice production increased significantly from 10.5 million metric tons (mmt) in 1995 to 17.63 mmt in 2016, with a productivity of 4.38 tons per hectare (19, 20). Per capita rice consumption (PCRC) has increased significantly over the last decade, with an average of 109.5 kg in 2015 (21). PCRC is used in estimating rice import requirements and setting rice self-sufficiency targets (22). About 10% of the annual consumption requirement is augmented from imports. In 2010 and 2011, the Philippines was the biggest rice importer globally, importing 2.38 million tons. Supplying enough rice for every Filipino exerts a lot of pressure on the domestic rice industry, especially with the constantly growing population, increasing at a rate of almost 2% per annum (18). PCRC has risen by 13% nationally and has risen in all regions. Central Visayas has the largest increase of 41%, while Calabarzon has the least increase of 4% (23). Most of the low-income families live in rural areas, spend more on rice, and have higher PCRC than high-income and urban people (8, 22). A dietary survey conducted by the Department of Science and Technology—Food and Nutrition Research Institute (DOST-FNRI) showed that rice intake was lowest among the highest-income households (1,120 grams per day) and highest among low-income households (1,300 grams per day) (2).

**Figure 1:**
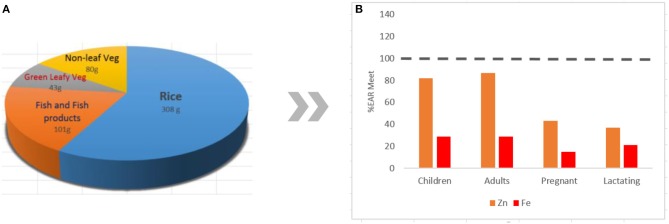
The typical mean daily dietary composition of Filipinos **(A)** and the hypothetical approximation of percent Estimated Average Requirement (%EAR) of Zn and Fe met for various population groups **(B)** (2). Bioavailability in the body is not considered in the approximation.

A typical Filipino diet is composed of rice-vegetable-fish combinations, with the highest percentage (37.2%) of rice and rice products in the total consumption and hypothetical approximation of percent Estimated Average Requirement (EAR) met for Zn and Fe for various population groups (Figure 1) (2). The diet can be characterized as high in carbohydrates, marginal in protein, and low in fat (8). Rice supplied the highest daily per capita calories, protein, and fats to Filipinos (Table 1) compared with other commodities (8). Rice, in its unpolished form, provides good amounts of nutrients such as vitamins B1, B6, and E; protein; minerals; unsaturated fat; and dietary fiber that can be found particularly in the embryo of the grain. The largest part of the grain, the endosperm, is mostly composed of carbohydrates with some incomplete protein and traces of minerals. On the other hand, the bran is mainly composed of carbohydrate cellulose, traces of vitamin B, minerals (including Fe, phosphorus (P), magnesium (Mg), and potassium (K), and incomplete proteins (23). The bran layers and majority (75–90%) of vitamins and minerals are removed during polishing to produce the commonly consumed white rice rich in starch but relatively low in micronutrient content (5, 23).

**Table 1:** Daily per capita calories, protein, and fat supplied by rice in the Philippine population from 2011 to 2017 (in grams).

**Nutrients**	**2011**	**2012**	**2013**	**2014**	**2015**	**2016**	**2017**
Calories	1139.08	1162.64	1130.94	1118.45	1097.99	1054.76	1156.51
Protein	23.93	24.43	23.76	23.50	23.07	22.16	24.30
Fat	5.74	5.86	5.70	5.64	5.54	5.32	5.83

*Source: (21)*.

Rice is a prime source of carbohydrate but it can also be a source of protein although it lacks several of the necessary amino acids required for good health; thus, it should be combined with other protein sources such as nuts, seeds, legumes, fish, or meat (26). Clearly, rice alone cannot provide the essential nutrients needed by the body to be healthy. The over dependence of Filipinos on rice as their main source of energy and nutrients, and the lack of diversity in diets, especially among the low-income class, make them vulnerable to various micronutrient deficiencies. Increasing the micronutrient concentration of rice will therefore have a significant impact on the nutritional status of rice-consuming Filipinos (11). A biofortified rice variety does not require additional input and can be grown by farmers in a sustainable way to produce micronutrient-dense rice grains that may be more accessible to nutrient-deficient populations, especially in agricultural areas (11).

## Importance of Iron and Zinc in Humans

The human body requires almost 50 known nutrients to carry out vital metabolic functions properly. Insufficient amounts of even one of these nutrients will lead to adverse metabolic disturbances resulting in poor health, impaired growth, and development in children, sickness, and eventually high economic losses to society (27, 28). This article will focus only on the importance and health consequences of deficiency in two micronutrients in review: Fe and Zn.

### Iron and Iron-Deficiency Anemia (IDA)

Iron acts as the central atom of hemoglobin (29) and a component of myoglobin (30), which functions in the storage of oxygen in muscle tissue, and of the cytochrome system (29), which is essential in the energy-releasing process of cellular respiration. In the body, it is present in the erythrocytes (60%), in readily mobilizable Fe stores (25%), and in myoglobin muscle tissue and in various enzymes involved in oxidative metabolism and other cell functions (31). Since Fe is involved in numerous and diverse cellular functions, a constant balance among iron uptake, transport, storage, and use is required to maintain Fe homeostasis in the body (32). Imbalances in the uptake or absorption and metabolic demand result in the depletion of Fe stores, which leads to deficiency (32).

Iron deficiency results when the body's supply of available Fe is too low. People with this condition cannot produce an adequate amount of hemoglobin to meet the body's oxygen-transport needs. The relationship between Fe deficiency and brain function is an important consideration in developing strategies to combat the former (33–35). The liver and brain have the same magnitude of Fe in their structures. The supply of Fe to the brain cells starts in the early phase of brain development; thus, early deficiency leads to irreversible damage to the brain (36). In humans, about 10% of the normal content of Fe is found in the brain at birth and this content reaches 50% at the age of 10 years old, with the optimum amount reached at the age of 20–30 years old. Fe deficiency can also cause immune system impairment, which renders the individual susceptible to infection (37, 38). When the deficiency becomes severe, the condition is diagnosed as IDA. The cut-off levels for moderate and severe anemia are 100 and 70 g/L (hemoglobin/blood), respectively. Common symptoms of IDA include tiredness and weakness and paleness in the hands and eyelids due to inadequate oxygen supply to the body's cells. Further, IDA can cause major health concerns in an individual at different life stages and at different ages. It can cause underdeveloped cognitive functions and poor physical coordination in children <2 years of age (which can be irreversible depending on the duration and severity), reduced physical endurance, and impaired immune system among other symptoms (39). It also affects memory, intelligence, and sensory perception, which directly impact academic performance and later influence economic productivity and earning potential (39, 40). IDA has also been shown to negatively influence the results of iodine supplementation (12). During pregnancy, IDA can cause irreversible changes to fetal kidney and neural development (41, 42).

### Zinc and Zinc Deficiency (ZnD)

Zinc plays essential roles in a wide variety of biochemical processes that affect growth, development, and reproduction and virtually all aspects of cellular metabolism (43). Zn has catalytic, structural, or regulatory roles and more than 300 mammalian enzymes are Zn-dependent (44, 45). Zn participates directly in enzyme catalysis or plays a role in the structural stability of proteins by supporting their folding and oligomerization (46). Further, Zn forms the prosthetic group of numerous enzymes as well as the receptor proteins for steroid and thyroid hormones and vitamins A and D (47). It also affects the function of enzymes involved in growth and development of bones (48). Zn enzymes are ubiquitous to all known classes of enzymes such as oxidoreductases, transferases, hydrolases, lyases, isomerases, and ligases that are involved in the synthesis and/or degradation of metabolic products such as carbohydrates, lipids, proteins, and nucleic acids (49).

Zinc deficiency (ZnD) results from an inadequate intake of absorbable Zn, an increase in Zn physiological requirements, inefficient absorption of Zn, increased losses, and impaired use of Zn. Serum Zn, the most available measure of ZnD, is used as an indicator of deficiency at the population level. The acceptable level of serum Zn is ~65 μg/dL (50). Severe deficiency can cause growth retardation and stunting, changes in neuro-behavioral development, impaired reproduction, and immune disorders (48, 51). It can also contribute to vitamin A deficiency (VAD) since the lack of Zn can reduce the synthesis of retinol binding protein (52). ZnD in a newborn or growing animal is considered fatal (48). Recently, global awareness of the importance of Zn nutrition to human health has increased dramatically. Although any age group can be at risk, infants, children, adolescents, and pregnant women are especially at risk because of their high requirements (53). Infants and young children have an increased requirement for growth (54). Adolescents reach the peak of physiological Zn requirement during the time of their pubertal growth spurt, which occurs in 10–15-year-old girls and 12–15-year-old boys (55). Pregnant and lactating women have increased Zn nutritional demands during these periods, especially during lactation (56). Elder persons often have an inadequate intake of Zn even in rich countries due to reduced consumption of Zn-rich foods and decreased efficiency of Zn absorption with age (31). Zn is an important nutrient for humans and its deficiency poses a significant widespread risk to human health. Age and sex are established variables that affect serum Zn content. Marginal caloric intake consisting primarily of starchy foods and low consumption of meat in the diets of older children contributes to increased risks of ZnD. On the other hand, the vulnerability of males to ZnD is largely attributed to higher growth rate and greater proportion of muscle per kilogram of body weight since muscles contain higher Zn content than fat (57).

## Iron and Zinc Status of the Philippine Population

Malnutrition is a large and growing problem worldwide with more than two billion people suffering from micronutrient malnutrition (58). It is estimated that 25% of pre-school children and 37% of non-pregnant women of reproductive age are Fe deficient (59). On the other hand, it is estimated that 17.3% of the world's population has low Zn intake, with the highest prevalence in Africa (23.9%) and Asia (19.4%) (60), contributing to global development challenges—eradicating hunger, reducing child and maternal mortality, and ending all forms of malnutrition (61)—and this can cause inter-generational consequences (27). Deficiencies of these micronutrients are concentrated in the semi-arid tropics, particularly in South and Southeast Asia and sub-Saharan Africa (62). Malnourished women may give birth to infants with low birth weight and greater risk of micronutrient deficiencies (61). Meeting nutritional requirements before and during pregnancy, at childbirth, and immediately post-partum positively impacts the survival, growth, and development of the fetus and newborn.

The 2015 Philippine National Nutrition Survey conducted by the Department of Science and Technology-Food and Nutrition Research Institute (DOST-FNRI) indicated that the mean per capita intake per day of meat and fish products, which are considered as the best sources of micronutrients, accounted for only 7.3 and 11.9% of the total daily intake, respectively. Meanwhile, a large percentage (70%) of the total daily intake of the Filipino diet came from plant sources, primarily rice and corn (maize) that contain high amounts of phytate, a potent inhibitor of micronutrient absorption (63).

### Iron-Deficiency Anemia (IDA) in the Philippines

Iron-deficiency anemia (IDA) is considered the most common form of anemia in the Philippine population (9, 64); hence, anemia is used as its proxy indicator. The highest prevalence of anemia in the country were observed in infants aged 6–11 months (40.1%), pregnant women (24.6%), elderly males (23.0%), elderly females (19.1%), and lactating women (16.7%) (65, 66). Rural areas have higher levels of anemia than urban areas (11.7 vs. 10.7%) (67). Moreover, the prevalence of anemia among children aged 6 months to 1 year from low-income families is considerably higher (16.5%) than among those from high-income families (7.9%) and this trend persists in older children (68). Anemia is more common among teenage pregnant women (30.6%) than among their adult counterparts (25.4%). More cases of pregnancy anemia were observed in urban areas (30.3%) than in rural areas (21.8%) (66).

IDA in pregnant women increases the incidence of infant mortality (the Philippines being among the countries with the highest rates in Southeast Asia), low birth weight, and premature birth (69). Although a substantial drop in the overall prevalence rate of anemia from 28.9% in 1993 to 11.1% in 2013 was observed, the prevalence rate among infants aged 6 months to <1 year is still at 40% and is considered a “severe” public health problem (66, 67).

IDA negatively affects the economy of the country in terms of “cost of illness” (which deals with health-care costs and productivity losses) and in terms of “burden of the disease,” which identifies the impact on the affected individual in terms of years lived with disability (disability-adjusted years; DALYs) and years of life lost (51). Weiser et al. (51) estimated the cost of illnesses due to IDA, VAD, and ZnD in two age groups (6–23 and 24–59 months) of Filipino children by socioeconomic strata. The total lifetime costs of IDA, VAD, and ZnD amounted to USD 30 million for direct medical costs, USD 618 million for production losses, and 122,138 DALYs for intangible costs. The cost of IDA is mainly due to its impact on the mental development of children aged 6 to 23 months, which translated into 100% production losses and 84% DALY losses. For 24- to 59-month-old children, additional effects of IDA are limited to DALY losses because of reduced physical activity. Production and intangible losses are also mainly contributed by IDA (51). A decrease in cognitive performance in children due to Fe deficiency also leads to a decrease of 4% in hourly earnings in their later life (69). The estimated decrease in gross domestic product (GDP) is as much as 2% per year due to widespread Fe deficiency in the country that could be translated into income losses of PHP 172 or 0.9% in severely stunted workers, and for workers engaged in moderate (5%) and heavy (17%) physical labor (61). The bulk of the costs incurred came from projected lifetime costs, costs resulting from impaired mental and physical development, and costs of premature death. Results of the study emphasized the importance of addressing micronutrient deficiencies in high-risk infants and young children belonging to the lowest-income households (51).

### Zinc Deficiency (ZnD) in the Philippines

ZnD ranks fifth among the top 10 leading illnesses and diseases in developing countries. In the Philippines, the first Zn status assessment study based on serum Zn level conducted by FNRI showed a high magnitude of prevalence of ZnD across population and physiological groups (63, 66). The occurrence rate of ZnD was high in school children (30.8%), male adolescents (32.2%), adults (31%), and lactating women (39.7%). The study covered 79 provinces in 17 regions, including the National Capital Region (NCR). It was observed that Zn-deficient pre-school and school children manifested a significantly higher rate of stunting than their normal counterparts. Stunting at an early age has been associated with poor cognitive ability (70) and increased child morbidity and mortality (71). A stunted child is also highly at risk for chronic diseases, fat-impaired oxidation, reduced glucose tolerance (31), and increased risk of severe infections such as diarrhea and acute respiratory infection (51, 72). Zn-deficient school children were also likely to have higher prevalence of anemia, VAD, and iodine deficiency. Anemia and stunting are the alternative indicators of ZnD (50).

ZnD has resulted in economic and human costs that have lifetime consequences, based on the study conducted by Weiser et al. (51). This study quantified the cost of micronutrient deficiencies (including ZnD) in the country. ZnD dominated the direct medical costs (89% of the total) because of the cost of treating diarrhea, respiratory diseases, and measles. Future production losses due to stunting brought about mainly by deficiency contributed 28% of the total production losses. However, compared with other micronutrient deficiencies (IDA and VAD), ZnD has lower intangible costs or DALYs mainly because of the absence of its impact on mortality (51). A systematic review of Zn supplementation trials showed that Zn had no significant effect on mortality (73).

Given the high prevalence of ZnD in the country that exceeds 20% (63), the International Zinc Nutrition Consultative Group (IZiNCG) recommended further assessment and planning for strategic intervention programs to be initiated (74). Hotz and Brown (74) suggested three intervention strategies: supplementation, fortification, and dietary diversification/modification. Current evidence indicates the beneficial effects of such interventions in reducing or eliminating the risk of ZnD (72). However, minimal efforts were carried out to control ZnD and resolve micronutrient malnutrition in the country (63).

Clearly, the impacts of IDA and ZnD on health and the economic sector of the country are alarming and require immediate attention. Furthermore, this highlights the importance of implementing intervention strategies to reach the vulnerable sectors of the population.

## Iron and Zinc Status of Philippine Soils

Micronutrients are essential in plant nutrition and production. They are required in minute quantities generally below 100 parts per million (ppm) in plant tissues (75) but are equally as important as macronutrients in plants. The relationship between micronutrient supply and crop growth has been examined and trace elements such as Zn, Mn, and copper (Cu) were shown to contribute to achieving higher yields (76, 77). Further, several studies suggest that the application of micronutrients to crops might result in more vigorous seedlings, increased disease resistance, and possibly improved drought tolerance (76, 78–80).

### Iron in Plants, Soil, and the Rice System

Iron is an important micronutrient in all living organisms since it plays an important role in numerous metabolic processes such as DNA synthesis, respiration, and photosynthesis (81). In particular, it is involved in the synthesis of chlorophyll and maintaining chloroplast structure and function in plants. The photosynthetic cells of plants contain ~80% of the total Fe in plants, emphasizing the important role of Fe in the electron transport system, biosynthesis of cytochromes and heme-molecules, and construction of Fe-S cluster assemblies (82). Essentially, the cytochromes act as electron carriers in the respiratory chain (83). Fe is a major component of plant redox systems because of its high affinity to active metalloprotein sites where it acts as a co-factor in redox reactions (84). For example, mitochondria contain a large amount of metalloproteins that require Fe to function (85). Several Fe-containing proteins are involved in both the respiratory chain and tricarboxylic acid cycle. Crucial steps in Fe-S cluster assembly for the entire cell take place in mitochondria, suggesting the importance of this compartment in Fe handling by the cell (86).

Fe is the fourth most abundant element in the lithosphere and is present in large quantities in the soil; however, it is also considered as the third most limiting nutrient in plant growth. Fe is predominantly found in the Fe^3+^ form mainly as a constituent of oxyhydroxide polymers whose very low solubility renders them insufficient for plant needs in aerobic environments (81, 87, 88) and alkaline soils (89). Deficiency in the soil is due to several factors: low concentration of soluble Fe^2+^ in upland soils; insufficient soil reduction under submerged conditions with low organic status; high-pH alkaline soils; excessive P fertilizer application; excessive concentrations of Mn, Cu, Zn, Mo, Ni, and Al in soil; and genotypes with low potential for excretion of organic acids to solubilize Fe (89). In contrast to ZnD, Fe deficiency is common in high-pH aerobic soils. Widespread Fe toxicity was observed in rice grown in wetland acid sulfate soils (90). However, Fe deficiency may occur in flooded soils when there is insufficient organic matter decomposition to drive the reduction of Fe^3+^ to Fe^2+^ and redox potential remains high. Fe deficiency in rice plants is manifested by interveinal chlorosis and yellowing of emerging leaves, poor root formation, and growth retardation (91). Since Fe is non-mobile within rice, symptoms initially appear on young leaves. In severe cases, the whole plant becomes chlorotic and dies. Fe deficiency is an important concern in dryland soils but it usually disappears 1 month after planting (89). Crops suffering from this deficiency have retarded growth and are more susceptible to diseases (16, 92). The deficiency of this nutrient leads to a decrease in dry matter production, chlorophyll content, and activity of enzymes involved in sugar metabolism.

Iron deficiency is a costly and difficult nutrient deficiency to correct. Unless applied in large amounts, inorganic Fe fertilizers are often ineffective in overcoming Fe deficiency. Deficiency is usually treated with the application of solid FeSO_4_ or Fe chelates through broadcasting, banding, or foliar applications (93). Applying organic fertilizers such as rice crop residues or manures as nutrient sources also helps alleviate Fe deficiency in the soil. A combination of foliar application of FeSO_4_ (3% solution) at different sowing intervals followed by soil application (150 and 305 kg FeSO_4_) was found to be an efficient and cost-effective method in improving Fe availability in aerobic rice soil conditions (94).

### Zinc in Plants, Soil, and the Rice System

Zinc is an essential micronutrient required for crop growth. Zn has chemical properties similar to those of other nutrient elements such as Fe and Mg. As such, competition between these elements can occur during absorption (95). Zn acts as an activator of many enzymes in plants and the metal component of enzymes or a functional structure, and regulatory co-factor of a large number of enzymes. It is also an essential component of RNA polymerase and a constituent of ribosomes, which is essential for their structural integrity (75). It is involved in several biochemical processes in the rice plant such as cytochrome (96) and nucleotide synthesis (97), chlorophyll production (96), and enzyme co-factor and activator, and it maintains membrane integrity (98–100) and auxin metabolism (101, 102). This micronutrient is bioavailable as Zn^2+^ in the soil solution or as exchangeable Zn on cation-exchange sites, organically complex Zn in solution, or organically complex Zn in soil solids. The normal exchangeable Zn extracted from the soil solution ranges from 0.05 to 0.25 ppm using water and ranges from 0.1 to 2 ppm using ammonium acetate. Lower values can be observed in soils where ZnD occurs in plants (103).

ZnD is common in rice soils, especially in anaerobic irrigated lowland fields, calcareous soils, and in rice soils with high pH and high organic content (75, 89, 103). The deficiency of this micronutrient in relation to flooded rice soil conditions has been well-documented (104, 105). Submergence in conventional flooding depletes soil oxygen, decreases redox potential, and increases pH, which eventually reduces the solubility of Zn in the soil (106). Meanwhile, low Zn concentration in calcareous soils is associated with high bicarbonate content and high pH (107) that inhibit translocation of Zn from roots to shoots (108). Commonly, transplanted seedlings (2–4 weeks) are susceptible to ZnD manifested by stunting, which eventually leads to delayed maturity and reduced yield (109). Zn and P ratio was observed to be related to the manifestation of ZnD. The interaction of Zn and P in the soil is highly affected by the infection of roots with vesicular arbuscular mycorrhiza on which infection of the roots facilitates absorption of Zn. However, increasing the supply of P in the soil strongly suppresses infection of the roots. The connection of P toxicity to ZnD is still not clear (110).

Plants grown in Zn-deficient soil tend to have stunted growth manifested by a reduction in stem length and shortening of internodes. Plant leaves exhibit rosetting, mottling, and interveinal chlorosis, and sometimes red spots appear due to the deposition of anthocyanin (75). Rice grown in Zn-deficient soils exhibits small brown spots and poor root development. Zn is one of the important micronutrients that affect growth and yield in rice and it has been an integral factor that determines the rice production of numerous countries. ZnD in soils is effectively corrected by Zn fertilization (89). It can be applied to soil through surface application or soil incorporation (105, 109) through seed treatment (111–114), foliar application (115–119), and by dipping seedlings into Zn solution (120–122) or in combinations. Numerous available Zn sources have been tested and studied individually or in combinations (103). Among the Zn sources, Zn sulfate is commonly used in rice and is usually applied through soil incorporation during flooding or after transplanting (89, 105). Although soil application has been proven to be an effective strategy in improving Zn concentration in tissues as well as improving yield in rice (123), it is not economically effective because of the high cost (103). Thus, other application methods or complementary approaches can be explored to maximize the availability of Zn in the soil at a lower cost.

Several agronomic approaches exist to improve the availability of Zn in rice production systems; these include tillage, crop rotations, and intercropping, manure application, fertilizer management (103), and water management. Franzluebbers and Hons (124) reported higher extractable P, K, and Zn in no-tillage land preparation vis-à-vis the conventional tillage system. However, Bhaduri and Purakayastha (125) recently reported that tillage operation has little effect on Zn availability in rice. On the other hand, crop rotation of rice with other crops such as wheat and maize has been reported to be beneficial in improving the availability of Zn in the soil such as the case of rice-maize rotation, which increases Zn fertilizer-use efficiency compared with continuously flooded rice (126). Manure application has also been reported to improve Zn availability in the soil by improving micronutrient availability through soil chemical, physical, and biological changes (127). This contributes to the accumulation of Zn under calcareous soils by reducing soil pH and improving mobilization of Zn. Furthermore, Azolla application in submerged soil conditions in rice also prevents ZnD by reducing bicarbonates in the soil (128). The availability of the Zn in the soil is highly affected by water regimes and aerobic conditions (129). Alternate wetting and drying (AWD) (130) has been proven to effectively facilitate absorption of Zn by increasing the redox potential of the soil. High concentrations of P in the soil create an antagonistic effect on the availability of Zn; hence, the application of large quantities of P fertilizers in the soil can induce deficiency in Zn and increase Zn requirements in plants (75).

### Micronutrient Soil Status of the Major Rice Areas in the Philippines

Limited information is available on the micronutrient status of Philippine soils since most of the soil fertility surveys conducted in the country focus only on the major macronutrients such as nitrogen (N), P, and K. The Bureau of Soils and Water Management (BSWM) of the Department of Agriculture (DA), the agency mandated to conduct soil surveys, mapping, and classification in the Philippines, is currently planning to conduct a soil fertility survey focusing on micronutrient levels of the country's soils (131). Previous accounts of the Zn-deficient soils in the country have been reported by Yoshida et al. (132) and these were estimated to be around 500,000 ha of irrigated rice. Katyal and Vlek (133) also reported ZnD in rice cultivated on Hydrosols (Gleysols) that caused severe yield reduction in plots not treated with Zn fertilizer. ZnD was observed in calcareous soils in the Philippines in which half of the 13 field trials conducted in the country showed deficiency in Zn (134). Alloway (135) also included the Philippines on the list of countries with widespread Zn deficiency in their soils. On the other hand, no previous survey of Fe-deficient areas in the country has been conducted.

A recent soil fertility survey was conducted by Magahud et al. (24) on paddy soils of major rice production areas in the Philippines in which they determined the concentrations of various nutrient elements in the soil and in the plants that included macronutrients such as K, Ca, and S and micronutrients such as Fe and Mn. The study reported toxic levels of the micronutrients Fe and Mn in most of the rice areas in Central Luzon, which was attributed to periodic soil submergence and increased soil acidity due to continuous cropping. The high concentration of Fe in San Leonardo, Nueva Ecija, was possibly due to the occurrence of Fe in clay-sized soil fractions in the area while high Fe and Mn in Sta. Cruz, Zambales, is caused by the input of mine wastes. Toxic concentrations of Fe and Mn (1,000 ppm and 300 ppm, respectively) were eventually observed in rice plants (24). It appears that Fe is highly available in rice; however, the bioavailability of this nutrient in the rice grain is poor (136).

**Figure 2:**
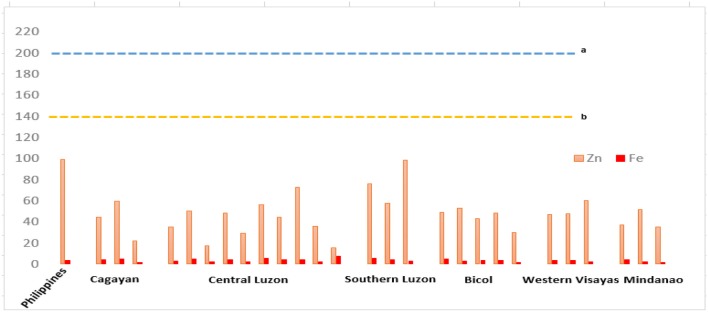
Micronutrient concentrations of major rice paddies in the Philippines (24, 25) in various regions in the country, with each bar representing different sample sites (provinces), showing deficiency in Zn based on (a) Canadian soil guidelines for agricultural soils and (b) Netherlands target values for soils. The concentration of nutrients is measured in ppm. Provinces in each region include Cagayan (Cagayan and Isabela), Central Luzon (Nueva Ecija, Tarlac, Bulacan, Pangasinan, and Zambales), Southern Luzon (Laguna), Bicol Region (Camarines Sur, Albay, and Sorsogon), Western Visayas (Iloilo and Negros Occidental), and Mindanao (Maguindanao, North Cotabato, and Sultan Kudarat).

In another study conducted by Magahud et al. (25), which assessed the levels of heavy metals, including Zn, in the country's major rice production areas found that Zn concentrations are generally low in rice production areas. Figure 2 presents the results of the studies on the concentrations of micronutrients, particularly Fe and Zn. The prevalence of ZnD in the rice-growing provinces was previously reported by Descalsota et al. (137) using soil samples collected from major rice areas in the country and identified the deficient elements in various provinces. The results indicated that all of the sites sampled were deficient in Zn. Meanwhile, sufficient concentrations of the micronutrients Ni and Mo were found at all of the sites sampled whereas few sites exhibited deficiency in Cu, particularly in Muñoz, Llanera, and Zaragosa in Nueva Ecija; La Paz in Tarlac; Sta. Cruz in Zambales; Casiguran in Sorsogon; and Zara in Iloilo.

## Co-Location of Zn Deficiencies in Philippine Soils and Population

Micronutrient deficiencies in the soil will produce micronutrient-deficient crops that provide insufficient amounts of micronutrients to humans, consequently contributing to micronutrient deficiencies in the population. There are strong indications of a causal relationship between micronutrient content in the soil and human micronutrient status (138). In order for crops to provide the necessary micronutrients for humans, they must obtain sufficient amounts of micronutrient initially from the soil. In rice, the environment, particularly the soil, has a significant impact on Fe concentration in rice grains (11, 134). It has been estimated that, among the agricultural soils in the world, 49% are deficient in Zn, 31% in B, 15% in Mo, 14% in Cu, 10% in Mn, and 3% in Fe. It appears that these micronutrient deficiencies are reflected in the human population. Among the micronutrient deficiencies, Bo receives less priority while Zn has the highest priority in human nutrition (136).

In the Philippines, a majority of the soils have multiple macronutrient and micronutrient deficiencies (24, 137). Zn is predominantly deficient based on the critical concentration (100 ppm for Zn) in the paddy soils where the survey was conducted (24). Zn-deficient paddy soils were identified for rice plants based on the results of the Minus-One Element Technique (MOET) followed by Descalsota et al. (137). A significant positive correlation and linear regression between soil Zn and plant Zn were observed by Magahud et al. (25), indicating that higher soil concentrations of Zn lead to higher Zn concentration in rice plants. The concentration of Zn in rice, particularly in the grains, is highly affected by the amount of bioavailable Zn in the soil (117, 130). The uptake of Zn in rice grains is suggested to be affected by the supply of Zn during the grain Zn loading period (139). The supply of Zn to the grains is via root uptake in Zn-sufficient conditions while grain Zn accumulation is via root uptake and remobilization in Zn-deficient conditions (111, 112, 140). Zn uptake and transport are the major bottlenecks for Zn biofortification in rice (141).

**Figure 3:**
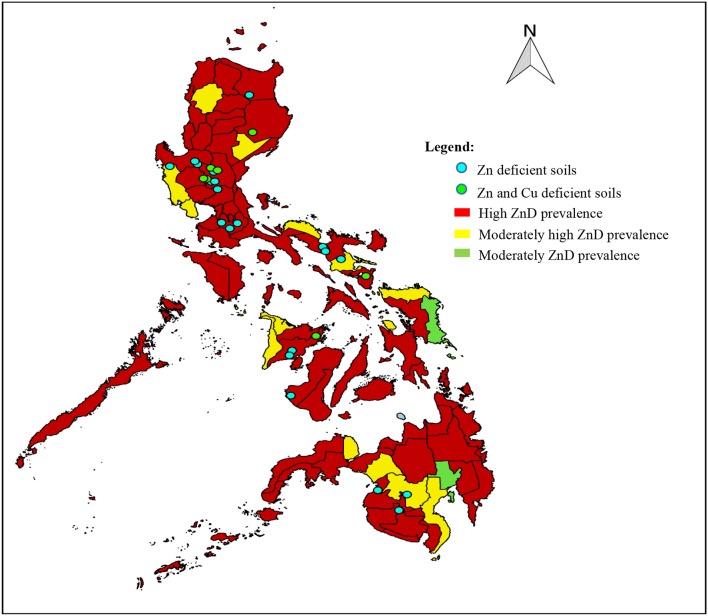
Locations of Zn-deficient soils and ZnD occurrence across age group populations in the Philippines. Sources: (24, 57, 137).

As stated earlier in this review, ZnD was observed across age groups and was widespread in all population groups, with overall prevalence of more than 20% (63). The widespread ZnD in paddy soils somehow corresponds to the incidence of ZnD observed in the populations that grow crops in the same soils. Figure 3 shows the overlapping locations of Zn-deficient soils and Zn-deficient populations in the country. Most of the areas that exhibited high occurrence of ZnD in the soil have ZnD in the population as well. The Zn-deficient soils where soil sampling was conducted represent the major rice production areas in the country and were also located in rural areas where the populations have a high prevalence of micronutrient deficiencies. Moreover, it can be observed that ZnD in both soils and populations is widespread throughout the Philippines. This is similar to the case in China, where micronutrient-deficient soils are located in areas of intensive agricultural production with high population density (116). Some areas exhibited both Zn and Cu deficiencies in the soils. Of all the micronutrients, Zn is directly linked to the food chain since deficiency in both humans and food crops is extensive (136). This observation needs further investigation to establish the relationship among micronutrient contents of soil, plants, and the human population in the country. A comprehensive evaluation of soil micronutrient contents and the development of a soil micronutrient map would be useful for this undertaking.

## Government Programs that Address Micronutrient Deficiencies

The Philippines has a Philippine Plan of Action for Nutrition (PPAN) as an integral component of the Philippine Development Plan for 2017–2022. It aims to expand and increase access to economic opportunities, especially for food for poor households, and reduce inequality in human development outcomes, as described in “Ambisyon 2040,” the country's 25-year development plan. The target outcomes of the PPAN are to reduce wasting from 7 to < 5%, reduce stunting from 33.4 to 28%, reduce micronutrient deficiencies, and have no further increase in overweight. Nutrition-specific programs, nutrition-sensitive programs, nutrition-supportive programs, and nutrition-enabling programs that involve the active participation of local government units are planned. Specific programs to combat micronutrient malnutrition include micronutrient supplementation and mandatory food fortification, that is, flour fortification with Fe and vitamin A, cooking oil fortification with vitamin A, sugar fortification with vitamin A, salt iodization, and rice fortification with Fe (142).

Although numerous intervention programs are currently implemented to alleviate the problems in micronutrient deficiencies, awareness of these programs poses a major challenge. A recent survey conducted by FNRI on various age groups (13–59) across the country revealed that, out of 80,064 respondents, only 8.2% were aware of micronutrients, their deficiencies, and clinical outcomes. Nutritional campaigns and public health awareness targeting highly at-risk groups need to be strengthened to effectively achieve the objectives of the government programs.

Tables 2, 3 list the government policies and intervention programs aimed at reducing micronutrient deficiencies, general malnutrition, and other health-related problems in the country.

**Table 2:** Government programs that address malnutrition in the Philippine population.

**Programs**	**Target population**	**Nutrients provided**	**Sources**
Pantawid Pamilyang Pilipino Program (4Ps)	Children and pregnant women	General nutrition	(143)
PhilHealth	All	General nutrition	(143)
Expanded Program on Immunization (EPI)	Infants	Vaccine	(143)
Food fortification	Infants, children, and adults	Fe, Zn, iodine, vitamin A, lysine	(9, 41, 60, 144)
Vitamin A supplementation	Children	Vitamin A	(143)
Fe supplementation	Young children and pregnant women	Fe	(61, 66, 143)
Multi-Nutrient Rice Kernel (MNERK)	All	Fe, Zn, vitamin A, B_9_, B_12_, B_1_, and iodine	(66)
Micronutrient Powder (MNP)	Children	Fe, Zn, and vitamin A	(143)
Gulayan sa Paaralan	Children	General nutrition	(143)
Rice biofortification	All	Zn and Fe	(16, 79, 145)

**Table 3:** Government policies crafted to combat malnutrition in the Philippines.

**Policies**	**Objectives**	**Target population**	**Program/service provided**	**Sources**
Philippine Plan of Action (PPAN) for 2017–2022	Address nutritional problems such as stunting, wasting, nutrient deficiencies, hunger, food security, and maternal nutrition	General population, especially children and pregnant women	General nutrition	(146)
DOH Memorandum No. 2011-0303: Micronutrient powder supplementation	Reduce under-five mortality rate by 66% and maternal mortality rate by 75%	Children and pregnant women	Vitamin and mineral supplements	(147)
Revised policy on child growth standard, 2010	Reduce undernutrition among children and compare growth of children against international standard	Infants and children	General nutrition	(148)
Philippine code on marketing and breast milk substitute (Revised 2010)	Support provision of safe and adequate nutrition of infants through protection and promotion of breastfeeding	Infants	Proper use of breast milk substitutes and supplements	(149)
Revised policy on micronutrient supplementation	Address micronutrient deficiencies	Children, pregnant women, and lactating women	Micronutrient supplements	(150)
Strategy for maternal, newborn, and child health and nutrition	Provide strategy to reduce maternal and neonatal mortality	Pregnant women and infants	Integrated maternal, newborn, and child health	(148)
Philippine Fortification Law (R.A. 8976)	Compensate inadequacy of micronutrients in the Filipino diet	All	Philippine food fortification program	(151)
Early Childhood Development Act, 2000 (R.A. 8980)	Promotes the rights of children to survival, development, and special protection	Children	National system for childhood care and development	(150)

### Government Policies

The Philippines is regarded as a “strong” nation in nutrition governance (152) and it has signed on to the Scaling Up Nutrition (SUN) Movement (146) to show its commitment to alleviating the problem of malnutrition. The Philippine government has crafted numerous policies and laws to address malnutrition and micronutrient deficiencies in the country (Table 3).These policies aim to reduce micronutrient deficiencies in the population by prioritizing the high-risk groups (infants, children, and pregnant and lactating women) through the introduction of various strategic intervention programs such as fortification, supplementation, and diversification. The government's initiative to eradicate micronutrient deficiencies, particularly Fe deficiency by enhancing Fe content in rice (Fortification Law), has paved the way for researchers to introduce alternative strategies to significantly increase the micronutrient content in rice through biofortification.

### International Support

International agencies such as United Nations International Children's Emergency Fund (UNICEF), World Health Organization (WHO) (153), Food and Agriculture Organization (FAO) are staunch supporters in combating malnutrition in the country. In particular, UNICEF has actively advocated and promoted nutritional fortification of food to combat micronutrient malnutrition, iodization of food grade salt, pushed breastfeeding in the first 6 months of life, and paved the way for vaccination, Vitamin A supplementation and deworming of children (154). On the hand, FAO complemented government's efforts through technical and development interventions in the field of agriculture, fisheries, forestry and rural development (155). WHO aims to help the country reach every citizen with health coverage based on international standards. Supplementary Table 1 presents some of the programs, projects, and collaborative efforts with international agencies to support Philippine government in tackling malnutrition.

## Healthier Rice varieties: Biofortification Efforts in the Philippines

Developing rice varieties with high micronutrient concentration within the grains is a cost-effective and sustainable strategy to address micronutrient needs among populations unable to access a healthy and diverse diet. Food fortification, supplementation, and diet diversification have been suggested strategies to combat micronutrient malnutrition (156) but they have been found to have varying degrees of success in addressing micronutrient deficiencies in developing countries (12, 13). Biofortification is a complementary solution that offers numerous advantages compared with other fortification strategies such as accessibility for malnourished individuals in rural areas. It is cost-effective, low-cost, and sustainable (157). A biofortified rice variety can be grown indefinitely and the micronutrient-dense grains can be produced in a sustainable way without any additional input to reach malnourished populations. Biofortification has no marginal cost compared with fortification and supplementation that require continuous financial outlays. Further, biofortified rice varieties can be evaluated and adapted to other locations, which multiplies the initial investment (158). The Copenhagen Consensus ranked biofortification and other interventions in reducing micronutrient deficiencies as the highest value-for-money investments for economic development (159).

The large genetic variability in rice germplasm for Zn micronutrient concentration makes it a suitable crop for biofortification through conventional breeding. Similarly, rice germplasm has significant genetic variation for Fe content (11). Developing rice varieties with high Fe and Zn content through biofortification also targets improving agronomic traits and increasing yield. The *HarvestPlus* program objectives have set the guidelines for breeding toward high Fe and Zn content. *HarvestPlus* is a CGIAR initiative that spearheads biofortification efforts through an umbrella of international agricultural research centers in developing new varieties of vitamin- and mineral-rich staple foods (28). Biofortification breeding should maintain or improve crop productivity, increase micronutrient content to positively impact human nutritional status in a measurable way, ensure the stability of improved traits across environments and climatic conditions, test the bioavailability of micronutrients in biofortified lines in humans to ensure benefits to people preparing and consuming them in traditional ways within normal conditions, and test consumer acceptance to ensure maximum impact on nutritional health (160).

*HarvestPlus* has screened more than 7,500 rice lines and has identified promising donor lines in breeding for high-Zn rice varieties in other Asian countries, such as Bangladesh and India (161). The Bangladesh Rice Research Institute (BRRI) has released five conventionally bred high-Zn rice varieties: BRRI dhan62, BRRI dhan72, BRRI dhan64, BRRI dhan74, and BRRI dhan84 (162). Meanwhile, the ICAR-Indian Institute of Rice Research has released DRR dhan 45, a high-Zn rice variety with 22.6 ppm Zn content in grains in polished form (163). China has released a rice variety with high Fe content, Zhongguanxian 1, with 7 ppm Fe content in polished form, released in 2010 (164).

The International Rice Research Institute (IRRI), situated in Los Baños, Laguna, Philippines, has been evaluating rice germplasm for Fe content variability since 1992 (11) and started working on Zn in 1995 (165). This research effort was influenced by the Philippine government's initiative to eliminate Fe malnutrition in the country by artificially enriching rice with Fe (11). Initially, improved lines with enhanced grain Fe and Zn coupled with high grain yield and desirable agronomic traits were developed. The most popular line was IR68144-3B-2-2-3, a line developed from crossing IR72, a high-yielding line from IRRI, and Zawa Bonday, a tall traditional variety from India, which was identified as a donor parent for high Fe content in grains (11). Two years after the establishment of the regional iron rice biofortification program (2003), NSIC Rc172 (MS13), tagged as the first high-Fe rice variety, was released. In 2016, the first zinc-rich variety, NSIC Rc460, was released by the National Seed Industry Council (NSIC) for production, 14 years following the release of MS13. This variety is a product of collaborative efforts between IRRI and the Philippine Rice Research Institute (PhilRice).

**Figure 4:**
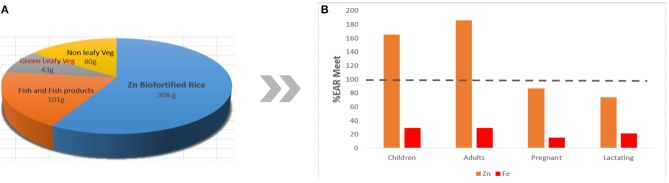
Hypothetical approximate %EAR met for Zn and Fe of various population groups **(B)** if rice is replaced with Zn-biofortified rice in the typical Filipino diet **(A)**, assuming that the target content of 28 ppm is achieved while bioavailability in the body is not considered in the computation.

The Department of Agriculture (DA) through PhilRice has been working closely with IRRI in developing micronutrient-dense elite lines. Breeding materials from both research institutes are assembled and evaluated, and field trials are conducted at various PhilRice stations and commenced in the last wet season of 2014. Line development at PhilRice has generated 24 crosses involving various donor parents containing high grain Fe or Zn with high yield ability, resistance to various pests, and good grain quality. Currently, there are 54 entries in Advanced Yield Trials (AYTs), 22 entries in Observational Yield Trials, 22 entries in Preliminary Yield Trials (PYTs), 43 entries in Pre-National Cooperative Testing (NCT), and 482 F_6_ lines being evaluated at different branch stations of PhilRice. Entries are composed of lines coming from both IRRI and PhilRice (166). As of the moment, two promising lines bred by PhilRice and six promising lines from IRRI are being evaluated in NCT and will be released later as varieties upon passing evaluation. The breeding target for biofortified Zn rice follows the target set by *HarvestPlus*, which was designed to meet the dietary needs of the population, and should provide 60–80% of the Estimated Average Requirement (EAR). Currently, the baseline Zn content of rice is at 16 ppm, with a final target content of 28 ppm (158). Figure 4 shows the approximate EAR met for Zn and Fe if rice is biofortified with Zn.

Breeding for rice varieties with enhanced nutritional content and desirable agronomic traits such as high grain yield are important breeding targets in rice (80). Development of high-yielding varieties with high level of Zn is doable (167). High Zn rice varieties with high-yield potential have been released for commercial cultivation in the Philippines and Bangladesh (162). Incorporating high Zn in hybrid rice can be a promising option. The current micronutrient content of hybrid varieties is relatively comparable with non-biofortified inbred varieties (168), increasing Zn content of hybrids can be an excellent breeding target. Several studies have revealed positive evidence on the possibility of developing hybrid rice varieties with high Zn content (23, 169). At IRRI efforts are being made to mainstream Zn breeding to incorporate grain Zn content as a key component of all the future rice varietal releases and also to develop hybrid varieties with high Zn content.

The limited number of micronutrient-enriched varieties available for farmers and consumers will be the bottleneck in the promotion of the varieties. The current released variety (NSIC Rc460) is still in the stage of seed multiplication and there are no recorded farmer users and areas planted to the variety. Awareness of the availability of this kind of variety and its purpose is still a major concern. The absence of an X-ray fluorescence (XRF) analyzer for evaluating the micronutrient content of promising lines delays the development of genotypes of PhilRice with high Zn and Fe content. The acquisition of an XRF analyzer is crucial for developing promising lines with high micronutrient content and an important factor in accelerating breeding programs for micronutrient-dense varieties.

## Future Prospects

Filipinos depend on rice as their main source of nutrition. The high dependence on rice and the lack of diversity in the diet have led to nutritional and health problems, particularly micronutrient deficiencies. Fe and Zn deficiencies are widespread and affect millions of Filipinos across ages and social strata with health and economic consequences. These consequences necessitate the design and evaluation of effective programs and policies for reducing micronutrient deficiencies.

Biofortification is an attractive strategy to enrich rice with micronutrients. It is an effective and sustainable approach that can target large portions of vulnerable populations. However, breeding efforts in the Philippines are slow. Currently, only two biofortified rice varieties, Zn rice, NSIC Rc460, and Fe rice, NSIC Rc172 (MS13), are commercially available for production and multiplication. However, a few more high-Zn rice varieties are in the final stage of testing in national trials and they will be released in the next 1 or 2 years. The availability of biofortified varieties that are high-yielding and suit farmers' preferences and consumers' taste will be the major prerequisite in effectively promoting biofortified rice to target populations. The limited options in the number of local varieties reduce the possibility of successful farmer adoption and delivery to target consumers. The lack of equipment for effective evaluation of grain micronutrient content of promising lines is the bottleneck in the development of varieties in local rice breeding programs.

Recently, the Department of Agriculture has been promoting the use of hybrid rice varieties under the High Yielding Technology Adoption (HYTA) program primarily due to their promising yield potential. Development of hybrid rice varieties with high micronutrient content can be a plausible option to address concerns on malnutrition and low yield. The program can be a perfect avenue to promote both technologies.

Once biofortified varieties are developed, the next step would be their promotion to farmers for production. Varieties are presumed to be tailored to farmers' preferences and, therefore, increasing awareness of their availability and their health benefits would be the next agenda. Nationwide farm demonstrations coupled with technology introduction during field days and gatherings would be effective means to promote varieties to farmers and seed growers as well as to increase use by consumers. Recently, the country has been actively advocating the consumption of brown rice through Presidential Proclamation No. 494 since this rice is more nutritious than polished rice and can potentially help to alleviate micronutrient deficiencies. The nationwide initiative of PhilRice and the DA in promoting the consumption of healthy rice such as brown rice can also be tapped in introducing other nutritious types of rice such as biofortified rice varieties. Furthermore, local governments can be tapped to issue ordinances and resolutions to support the advocacy of introducing and consuming healthy rice to rice stakeholders (170). The consumption of diversified food, especially other staples with higher micronutrients and vegetables, will be the ideal strategy, but an immediate change in diet and its availability and accessibility will be the limitation.

After biofortified rice has been introduced to the market and eventually to consumers, an impact assessment will evaluate the effect of consuming it on the micronutrient status of the target population. This could be similar to the study conducted by Haas et al. (171), which tested the efficacy of the additional Fe from the biofortified rice in the intake of Filipino women who are at risk of Fe deficiency. The study showed that 9 months of consumption of Fe-biofortified rice improved the Fe stores in the blood of non-anemic Filipino women (171). However, examining the bioavailability of micronutrients from plants to humans is difficult because of the governing interactions of various factors, particularly in determining the bioavailability of a particular micronutrient to an individual with a diversified diet (172, 173). The micronutrient status of test subjects affects the regulation of these nutrients within the body. It is suggested that, to maximize the response of subjects to a test meal, the subjects should be marginally depleted with micronutrients (172, 173). Ideally, human trials should be conducted in a controlled environment to generate reliable and conclusive data.

The locations of micronutrient-deficient populations, particularly with ZnD, coincide with the locations of Zn-deficient soils in the Philippines. It is crucial that breeding for superior genotypes be coupled with sound agronomic practices (such as Zn fertilizer application) that promote micronutrient availability in the soil to maximize micronutrient content in the grains. Increasing grain micronutrient concentration is not solely dependent on the genotype's capacity to accumulate micronutrients in its grains but is also dependent on soil micronutrient concentration. Philippine soils have been found to be Zn-deficient, especially in paddy soils. Deficiency in micronutrients in the soil leads to deficiency in plants, which eventually leads to lower amounts of micronutrients consumed by humans. It is therefore essential to consider the soil-plant-human system in optimizing micronutrient biofortification in rice that combines plant breeding and nutritional management of the soil-plant system with the aim of improving nutritional status in humans. This approach is more sustainable because it involves regulation of the micronutrient flow from soils to plants and eventually to humans. Further, interdisciplinary collaboration of plant breeders, plant nutritionists, soil scientists, and human nutritionists could result in the development of micronutrient-efficient and micronutrient-dense rice genotypes (116).

Breeding for biofortified rice rich in Zn and Fe should be a top priority in the Philippines and other rice-growing countries because it is a win-win approach since it is beneficial to both rice plants and human nutrition. Other approaches for biofortification, such as genetic engineering, should be explored, especially in the case of Fe since natural variability in this micronutrient is limited, particularly in polished rice. Lastly, the impact of biofortified rice can be realized through measurable improvements in the micronutrient status of target populations through community-based human feeding trials with a given duration.

## Author Contributions

AP, GD-E, and BS designed the review. AP wrote the whole manuscript. AA and MC wrote some parts of the manuscript. MI-A, EA, PC, TB, AL, JH, CA, RR, and BS edited and contributed significant inputs.

### Conflict of Interest Statement

The authors declare that the research was conducted in the absence of any commercial or financial relationships that could be construed as a potential conflict of interest.
