# Supplementary material for: Zinc and Iron Nutrition Status in the Philippines Population and Local Soils
Source: Front Nutr. 2019 Jun 7;6:81. doi: 10.3389/fnut.2019.00081 (PMC6568233; doi:10.3389/fnut.2019.00081)
Supplement: Supplementary file 1 [file Table_1.docx]

Supplementary Table 1. Initiatives, projects, supports, and collaborations with international agencies and national government that address malnutrition in the country.

| **Initiatives, programs, and supports** | **Target Population** | **Partner Agency** | **Source** |
| --- | --- | --- | --- |
| Super Snacks | All | UNICEF | [154] |
| Baby Friendly Hospital Initiatives | Infant | UNICEF | [154] |
| Country Program for Children | Children | UNICEF | [154] |
| Promotion of Milk Code | Infant | UNICEF | [154] |
| Advocate Iodisation of Salt (Asin Law) | All | UNICEF | [154] |
| Exclusive Breastfeeding | Six month old baby | UNICEF | [154] |
| Vaccination, Vitamin A supplementation, and deworming in conflict areas | Children | UNICEF | [154] |
| Early childhood care and development | Six years old and below | UNICEF | [154] |
| Marawi Support | Mothers and children | UNICEF | [154] |
| Strengthening and development and use of Rice Integrated Cropping Management (RICM) for food security and poverty alleviation | All/Farmers | FAO | [155] |
| ]Ensuring Food Security and Nutrition for Children 0-2 years old in the Philippines | Children (0-2 years old) | FAO | [155] |
| Livelihood Restoration and Improved Food Security of Internally Displaced People in Conflict Affected Communities in Mindanao | All | FAO | [155] |
| Exclusive Breastfeeding | First six months/Lactating mother | WHO | [153] |
| Essential Intrapartum and Newborn Care (EINC) | Infant/Mother | WHO | [153] |
